# Influence of Disease Knowledge on the Metabolic Control of Diabetes Mellitus: A Cross-Sectional Study

**DOI:** 10.7759/cureus.75439

**Published:** 2024-12-10

**Authors:** João O Marques, Andreia Bandeira, Roberta B Parreira, Adriana M Ferreira, Catarina C Roteia

**Affiliations:** 1 Esporões Family Health Unit, Braga Local Health Unit, Braga, PRT; 2 Gualtar Family Health Unit, Braga Local Health Unit, Braga, PRT; 3 Ruães Family Health Unit, Braga Local Health Unit, Braga, PRT; 4 Saúde Oeste Family Health Unit, Braga Local Health Unit, Braga, PRT

**Keywords:** diabetes knowledge test, diabetes mellitus, health literacy, primary health care, self-management

## Abstract

Aim: Diabetes mellitus is a prevalent disease in the Portuguese population and is associated with significant morbidity and mortality. Its proper therapeutic management is multifactorial, with lifestyle habits having a major impact. Studies show that poorer metabolic control is associated with deficient knowledge related to diabetes, lower self-efficacy, and limited patient empowerment. The aim of this study was to characterize diabetic patients’ knowledge about their disease using the Diabetes Knowledge Test (DKT) and to assess the potential correlation between disease knowledge and metabolic control.

Design: A cross-sectional study was conducted involving a convenience sample of diabetics followed in four primary care units in Braga, Portugal. The sample was divided into two groups: insulin-treated (IT) and non-insulin-treated (NT).

Methods: Each participant was given the DKT. Frequency measures were used to describe the sociodemographic characteristics and clinical parameters. For association between categorical variables, the Fisher's test and chi-square test were used. To compare the distribution of the response variable (metabolic control represented by glycated hemoglobin (HbA1C) values) between IT and NT, the Mann-Whitney test was used. For association between nominal qualitative variables, the chi-square test was employed. The statistical significance level used in the tests was 5% (p<0.05).

Results: A total of 99 responses were obtained, with an average age of 65.5 years. Most of the respondents were male, had a low education level, were overweight, and had type 2 diabetes diagnosed 10 or more years ago, with an average HbA1C value of 6.83%. The average performance obtained by the DKT scale revealed medium levels of knowledge (IT and NT). Both groups displayed a medium level of knowledge (59% for NT and 62.5% for IT), without statistically significant difference. There was a statistical difference in metabolic control between IT and NT (Mann-Whitney test, p<0.05). Regarding the degree of knowledge of the disease (by DKT results), there was no statistically significant association, using the Fisher's test (p=0.20), between the two groups. In NT, there was no statistically significant association between metabolic control and the degree of knowledge (Fisher's test, p=0.69). There was an association between metabolic control and the duration of the disease (Fisher's test, p=0.029, odds ratio 3.83) in NT, as in IT (p=0.025). However, there was not a proven association between metabolic control and the degree of knowledge in IT (Fisher's test, p=0.62).

Conclusions: This study provides a comprehensive analysis of the characteristics of patients with diabetes and the factors affecting both metabolic control and disease knowledge. Although general knowledge of diabetes was "medium" for most patients, this level of knowledge may not be sufficient to ensure good self-care, especially in IT and those with a longer duration of disease. A longer duration of diagnosis was associated with poorer metabolic control, which may be related to the natural progression of diabetes and the challenges in maintaining adequate control over time. This finding highlights the need for continuous and personalized strategies for managing the disease as it progresses. Furthermore, these results emphasize the need for public health policies that promote enhanced diabetes education, aiming to empower patients to manage their condition more effectively.

## Introduction

Diabetes mellitus (DM) is a chronic endocrinological disease that involves chronic states of hyperglycemia caused by abnormalities in metabolism, especially carbohydrates, resulting from the secretion and/or inappropriate action of insulin [1]. This disease can be associated with important comorbidities and complications, particularly in the kidney, nervous system, heart, circulatory system, and eye, and the possibility of these occurrences can be minimized and controlled through adequate metabolic control of the disease and regular multidisciplinary clinical follow-up [2]. Currently, it is estimated that DM has a worldwide prevalence of approximately 9% [1]. It is expected that this prevalence will increase in the coming years and that it could exceed 10% by 2040 [3]. Among the different types of DM, type 2 diabetes mellitus (DM2) represents around 90-95% of all cases worldwide [3].

In the PREVADIAB study (2010), the total prevalence (diagnosed and undiagnosed) of DM, in the Portuguese population aged between 20 and 79 years, was 11.7% (6.6% diagnosed and 5.1% undiagnosed) [4]. In the 2019 edition of the annual report of the National Diabetes Observatory, the prevalence of DM in the Portuguese population (between 20-79 years old) was already higher, with an estimated 13.6% of them having diabetes (7.7% diagnosed and 5.9% undiagnosed). In this report, there was also a worrying prevalence of intermediate hyperglycemia states (impaired fasting glycemia (IFG) and impaired glucose tolerance (IGT)) in the Portuguese population between 20 and 79 years old, with 10.6% presenting only IFG, 14.6% only IGT, and 2.8% IFG associated with IGT, calculating that “41.6% of the Portuguese population (20-79 years) has diabetes or intermediate hyperglycemia” [5]. This is a multifactorial disease, in which there is a key role played by habits and lifestyles that directly impact both the risk of developing the disease and its adequate control. It is known that it is crucial that patients with diabetes have a good knowledge of the disease, its conditions, and lifestyle measures capable of influencing control and outcomes associated with DM. It is essential, in terms of habits and lifestyles, that the user can adopt these behaviors daily with a view to better controlling their disease. Therefore, it seems unusual that these patients commonly present difficulties in adopting dietary choices appropriate to their disease [1].

Measuring glycated hemoglobin (HbA1C) is the most efficient way to assess the metabolic control of diabetes as this value indirectly reflects the average glycemic value of the last three months. Its measurement is recommended both in the evaluation of therapy and in the control of diabetes and should be dosed every six months in patients within the therapeutic target or every 3-4 months, or whenever necessary, in patients who have undergone therapeutic adjustment or who are still not within the therapeutic target. However, as HbA1C is an indirect measure of the average blood glucose value, it has some limitations related to its variability due to, for example, conditions that affect the turnover of red blood cells. Therefore, this value may represent an under or overestimated value of the average glycemia and the doctor must consider these factors when evaluating metabolic control. In patients with DM2 under insulin therapy or in patients with type 1 diabetes mellitus (DM1), HbA1C is not a faithful representation of glycemia variability, so in these patients, metabolic control must be interpreted together with capillary blood glucose values ​​and/or continuous blood glucose monitoring. The therapeutic target recommended by the American Diabetes Association for most adult diabetics (except for pregnant women) is an HbA1C of 7%. This value must be adjusted depending on individual factors of a given patient, and the adoption of more demanding targets (i.e., HbA1C values ​​<7%) is justifiable if achieved safely and without risk of hypoglycemia or other treatment effects) or more liberal values ​​(i.e., HbA1C values ​​<8%, especially in patients with shorter life expectancy) [6].

A 2019 systematic review of the factors associated with glycemic control in patients with diabetes allowed us to understand these factors and group them into three large groups: sociodemographic factors, clinical characteristics, and factors associated with the user. Regarding the sociodemographic factors addressed (age, ethnicity, and socioeconomic factors such as income, education, and professional status), no consistent findings were identified that could conclude a relationship between age and metabolic control. However, regarding socioeconomic factors, this review reported that evidence suggests a positive association between a higher socioeconomic status and better metabolic control. This review concluded that the evidence regarding the association between disease duration and metabolic control is unclear. In terms of medication use, it was found that poor self-management of insulin regimens - either due to lack of knowledge or insufficient technical skills for therapeutic adherence - correlated with worse glycemic control. This work also noted that the presence of comorbidities associated with diabetes represents a greater challenge in glycemic control. Regarding body mass index (BMI), the data presented in this review were incongruous, indicating that both diabetics with high and low BMI values ​​may have poor metabolic control, thereby suggesting that the interpretation of BMI must be incorporated into a holistic understanding of the state of individual's health so that it can be correlated with metabolic control. Finally, regarding patient-centered variables, this systematic review demonstrated consistent evidence of the association between worse metabolic control and poor knowledge related to diabetes, with lower levels of self-efficacy and little user empowerment [7].

Health literacy can be defined as the ability of a given individual to receive or obtain, assimilate, and adequately understand information necessary for health decision-making, as well as their ability to access and orient themselves within health services [8]. Some individual skills, such as reading, writing, numeracy, communication, and the ability to use technological means, are inherent to health literacy [8]. It is estimated that more than 30% of the American population has a basic or lower level of health literacy and that, even among those who have an intermediate level, there are clear difficulties in understanding and carrying out some simple tasks related to health, such as understanding dosage instructions for prescribed medication [8]. Low levels of health literacy can translate into worse health outcomes and a less responsible and efficient use of health resources [9]. The population of individuals with diabetes is a special example of the need for good health literacy indicators as they play an active and daily role in controlling their disease: through the habits and behaviors adopted, through understanding their disease, or through adequate therapeutic management (which includes the management of insulin therapy regimens that require great understanding and capacity on the part of the patient), as well as the need for regular medical follow-up. Diabetics with lower levels of health literacy are, therefore, at greater risk of developing comorbidities and complications associated with their disease [9]. Caruso et al. (2018) conducted a systematic review of systematic reviews on health literacy in patients with DM2 and concluded that there were some areas of consensus and some gaps in the literature on the evidence regarding health literacy in patients with DM2. The areas of consensus mentioned were the definition and measurement tools of health literacy, as well as the influence of a patient's knowledge on their level of literacy. The gaps mentioned were related to the influence of the environment and gender, the cost-effectiveness analysis of interventions to increase health literacy, the effectiveness of interventions to increase health literacy, the relationship between health literacy and self-efficacy, and the relationship between health literacy and health outcomes [3]. In the meta-analysis by Marciano et al. (2019) on the role of health literacy in knowledge about diabetes, self-care, and glycemic control, a statistically significant association was found between high levels of health literacy and better knowledge about DM; high levels of health literacy and greater frequency of self-care activities (such as adopting an appropriate diet, physical exercise, therapeutic adherence, foot care, among others); and high levels of health literacy and better metabolic control (that is, lower HbA1C values). This meta-analysis concluded that there is a modest but significant effect between health literacy and better glycemic control [2]. Another systematic review and meta-analysis by Kim et al. (2016) concluded that health literacy interventions on diabetes management are effective in improving glycemic control [9].

The Diabetes Knowledge Test (DKT) was developed by the Michigan Diabetes Research and Training Center with the aim of assessing the knowledge that patients with diabetes have about their disease, and this is a duly validated tool [10]. It was subsequently revised and updated in 2016, and its reliability and validity were verified [10]. The DKT can be applied equally to type 1 and type 2 diabetics. It consists of 23 questions and can be divided into two sections: first (corresponding to the first 14 questions) on general knowledge about the disease and second (last nine questions) with questions about insulin therapy (aimed at people undergoing insulin therapy, whether people with DM1 or DM2), making it possible to use these two sections independently [10]. The DKT is, therefore, a quick and inexpensive method for assessing knowledge about diabetes in the areas of general knowledge and self-care [11]. The psychometric systematic review by Montagut-Martínez et al. (2021) on assessment instruments for knowledge about diet in diabetes concluded that the DKT is one of the recommended instruments for this purpose in people with DM1 or DM2 [1]. There is no standard interpretation of the numerical result obtained by applying DKT; however, some duly applied examples can be found in the work of Zowgar et al. (2018) and Mufunda et al. (2012) [11,12]. A study carried out in Saudi Arabia, in which the DKT was applied to 942 individuals with diabetes, revealed that 66.1% had average levels of knowledge (defined as average knowledge obtaining 12-18 correct answers when applying the DKT in its entirety) and 4.7% high levels of knowledge (defined as high knowledge obtaining 19-23 correct answers when applying the DKT in its entirety). This study also verified the association between better levels of knowledge with a younger age, a higher level of education, a longer diagnosis of diabetes, and a family history of diabetes [11]. The verification, at the population level, of the reliability of DKT in Portugal was verified and proven by Azevedo and Santiago (2016) [13].

With the present study, the authors intended to try to characterize the knowledge that patients with diabetes have about their disease and its appropriate management through the application and interpretation of the DKT questionnaire validated for the Portuguese population and, if possible, evaluate the eventual correlation between the results obtained by applying the questionnaire and the metabolic control of the disease.

## Materials and methods

This study was designed and implemented as an observational, analytical, and cross-sectional study. A convenience sample was drawn from diabetes patients at the healthcare units involved, with eligible participants being opportunistically invited to take part in the study. The eligibility criteria for participation in the study were as follows: diabetic patients (active coding T90 - Non-insulin-dependent diabetes or T89 - Insulin-dependent diabetes from the 2^nd^ International Classification of Primary Care (ICPC-2)), aged 18 years or older, followed at the Esporões Family Health Unit (USF), Gualtar USF, Ruães USF, and Saúde Oeste USF of the Braga Local Health Unit (Portugal), who voluntarily and informedly consented to participate in this study. Patients who met these conditions but had cognitive impairments or were unable to communicate/respond independently were excluded. Sample recruitment and data collection occurred after the approval of the study by the involved healthcare units, following a positive opinion from the Ethics Committee of the Braga Local Health Unit, and after obtaining informed consent from the participants. The data collected were strictly necessary for the development of this study and its objectives. No personally identifiable information about the participants was used. The collected data were pseudo-anonymized, with only the researchers having access to them. Once the study is completed and the results presented, all data will be permanently deleted. Data collection was conducted by the researchers in their respective healthcare units during medical consultations.

Demographic and clinical data were obtained through a clinical interview or by consulting the electronic medical records in the SClínico® program (SPMS EPE, Lisbon, Portugal). Data collection was conducted using a questionnaire created by the researchers, which included the variables under study as well as questions from the Diabetes Knowledge Test (DKT).

The variables under study included: age (in years), age group (18-24 years; <25 years; 26-35 years; 36-45 years; 46-55 years; 56-65 years; 66-75 years; ≥75 years), gender (Male; Female; Other/Not specified), education level (No formal education; Up to 4^th^ grade (inclusive); Up to 6^th^ grade (inclusive); Up to 9^th^ grade (inclusive); Up to 12th grade (inclusive); Vocational training; Higher education; Other), marital status (Single; Married/common-law partner; Divorced; Widowed; Other), employment status (Student; Unemployed; Active worker; Retired), type of diabetes (Type 1 diabetes; Type 2 diabetes; Unknown), time since diabetes diagnosis (<10 years; ≥10 years; Unknown), antidiabetic therapy (Insulin; Oral hypoglycemic agents (tablets) or glucagon-like peptide 1 (GLP-1) injectable analogs), therapeutic adherence (Adhere; Adhere with occasional forgetfulness; Adhere with frequent forgetfulness; Do not adhere/do not take medication), weight (in kilograms), BMI, HbA1c value in the last 6-12 months (if multiple values were available during this period, the arithmetic mean was considered), microvascular and macrovascular complications (No complications; Only microvascular; Microvascular and macrovascular), DKT result - 14 questions/non-insulin-treated (Poor knowledge (<7 points); Moderate knowledge (7-11 points); Good knowledge (>11 points)), and DKT result - 23 questions/insulin-treated (Poor knowledge (<11 points); Moderate knowledge (11-17 points); Good knowledge (>17 points)).

A bibliographic search was conducted in PubMed and the Index of Portuguese Medical Journals using the terms "diabetes knowledge test", the MeSH terms "diabetes mellitus" and "health literacy" with the Boolean operator AND (diabetes mellitus AND health literacy), and the terms "diabetes", "prevalence", and "Portugal" with the Boolean operator AND (diabetes AND prevalence AND Portugal).

Statistical analysis was performed using IBM SPSS Statistics software (IBM Corp., Armonk, USA). Initially, descriptive analysis of the data was conducted through the presentation of graphs and tables. Continuous quantitative variables were evaluated based on their mean frequencies (with corresponding standard deviations), and nominal qualitative variables were analyzed based on their absolute values and proportions. Additionally, the normality of the variables was assessed using the Shapiro-Wilk test. If the sample did not follow a normal distribution, statistical analysis was performed using non-parametric strategies.

For association between categorical variables, Fisher's test and chi-square were used. To compare the distribution of the response variable (metabolic control represented by HbA1C values) between the groups, the Mann-Whitney test was used. For association between nominal qualitative variables, the chi-square test was employed. The statistical significance level used in the tests was 5% (p<0.05).

## Results

The interview with diabetic patients and data collection took place from May 2024 to August 2024, with a total of 99 responses being obtained during this period. Descriptive statistics showed us that the typical patient who participated in this study had an average age between 65 and 66 years, was male, with a level of education up to the 4^th^ year of education, with a married/de facto relationship status, and was professionally active. This analysis also showed us that this type of user was overweight, had type 2 diabetes diagnosed 10 or more years ago, reported adequately complying with oral antidiabetic therapy, maintained metabolic control with an average HbA1C value of 6.83%, and a median of 6.45%, with no complications associated with diabetes. The average performance obtained by applying the DKT scale revealed only an average level of knowledge both in insulin-treated and non-insulin-treated patients.

Tables 1-2 descriptively present the frequencies and proportions for all participants and in the subgroups of non-insulin-treated and insulin-treated users for the sociodemographic variables and the variables relating to the disease, respectively. Table 3 presents the descriptive statistics regarding mean and median values of age, weight, BMI, and HbA1C.

**Table 1:** Descriptive table of results for sociodemographic variables.

	Total (n=99)		Non-insulin treated (n=83)		Insulin treated (n=16)	
Age group	n	%	n	%	n	%
18-25 years old	1	1%	1	1%	0	0%
26-35 years old	0	0%	0	0%	0	0%
36-45 years old	2	2%	1	1%	1	6%
46-55 years old	8	8%	8	10%	0	0%
56-65 years old	35	35%	28	34%	7	44%
66-75 years old	38	38%	33	40%	5	31%
≥76 years old	15	15%	12	14%	3	19%
Gender						
Masculine	62	63%	55	66%	7	44%
Feminine	37	37%	28	34%	9	56%
Education						
No education/illiteracy	1	1%	1	1%	0	0
Up to 4^th^ year of schooling (inclusive)	41	41%	32	39%	9	56%
Between 4^th^ and 6^th^ year of schooling (inclusive)	15	15%	13	16%	2	13%
Between 6^th^ and 9^th^ year of schooling (inclusive)	7	7%	6	7%	1	6%
Between 9^th^ and 12^th^ year of schooling (inclusive)	15	15%	13	16%	2	13%
Professional course	5	5%	4	5%	1	6%
Higher education (includes bachelor's degree, master's degree, doctorate)	15	15%	14	17%	1	6%
Marital status						
Single	5	5%	4	5%	1	6%
Married/de facto union	83	84%	71	86%	12	75%
Widower/widow	6	6%	3	4%	3	19%
Divorced	3	3%	3	4%	0	0%
Other	2	2%	2	2%	0	0%
Professional status						
Student	0	0%	0	0%	0	0%
Unemployed	0	0%	0	0%	0	0%
Active worker	41	41%	35	42%	6	38%
Retired	58	59%	48	58%	10	63%

**Table 2:** Descriptive table of results for variables related to the disease. GLP-1: Glucagon-like peptide 1

	Total (n=99)		Non-insulin treated (n=83)		Insulin treated (n=16)	
	n	%	n	%	n	%
Type of diabetes						
Type 1 diabetes	3	3%	0	0%	3	19%
Type 2 diabetes	96	97%	83	100%	13	81%
I don't know	0	0%	0	0%	0	0%
Duration of diagnosis						
<10 years	43	43%	40	48%	3	19%
≥10 years	56	57%	43	52%	13	81%
I don't know	0	0%	0	0%	0	0%
Therapy						
Oral antidiabetic	80	81%	80	96%	0	0%
Oral + injectable GLP-1 analog	3	3%	3	4%	0	0%
Oral + insulin therapy	15	15%	0	0%	15	94%
Insulin therapy	1	1%	0	0%	1	6%
Therapeutic compliance						
Does not comply/does not take medication	6	6%	4	5%	2	13%
I comply with few forgetfulness (<1 per week)	6	6%	4	5%	2	13%
I deal with a lot of forgetfulness (≥1 per week)	6	6%	4	5%	2	13%
Comply appropriately	81	82%	71	86%	10	63%
Complications of diabetes						
No complications	84	85%	75	90%	9	56%
Microvascular	7	7%	1	1%	6	38%
Macrovascular	3	3%	3	4%	0	0%
Neuropathic	2	2%	2	2%	0	0%
Neuropathic + microvascular	2	2%	1	1%	1	6%
Neuropathic + macrovascular	1	1%	1	1%	0	0%

**Table 3:** Descriptive statistics regarding mean and median values of age, weight, body mass index, and glycated hemoglobin value.

	Total (n=99)		Non-insulin treated (n=83)		Insulin treated (n=16)	
	Mean	Median	Mean	Median	Mean	Median
Age (years)	65.93	66.000	65.9	66.000	66.12	66.00
Weight (kg)	75.81	76.5	69.76	71.5	85.9	93.4
Body mass index (BMI) (kg/m^2^)	27.6	27.2	27.055	27	29.17	28.1
Glycated hemoglobin value (HbA1C) (%)	6.83	6.45	6.39	6.3	8.5	8.2

The results obtained by applying the DKT scale (Tables 4-5 for non-insulin-treated and insulin-treated groups, respectively) revealed an average level of knowledge in both the non-insulin-treated and insulin-treated groups (average score of 7.27 points and 13.56 points, respectively) according to the categorization of the degree of knowledge used by Mufunda et al. The group of non-insulin-treated diabetics answered only the first 14 questions of the questionnaire, with the results categorized as follows (one point was awarded for each correct answer): scarce knowledge <7 points; average knowledge if ≥7 and <11; and good knowledge if ≥11 points. Insulin-treated diabetics answered the 23 DKT questions, with the results categorized as follows: scarce knowledge <11 points; average knowledge if ≥11 and <17; and good knowledge if ≥17 points.

**Table 4:** Results obtained by applying the DKT questionnaire in the non-insulin-treated group. DKT: Diabetes Knowledge Test

Non-insulin-treated DKT result (14 questions)	Non-insulin treated (n=83)		Non-insulin treated (n=83)	
	n	%	Mean	Median
Scarce knowledge (≤7 points)	31	37%	7.27	7
Medium knowledge (7-11 points)	49	59%		
Good knowledge (≥11 points)	3	4%		

**Table 5:** Results obtained by applying the DKT questionnaire in the insulin-treated group. DKT: Diabetes Knowledge Test

DKT insulin-treated result (23 questions)	Insulin treated (n=16)		Insulin treated (n=16)	
	n	%	Mean	Median
Scarce Knowledge (<11 points)	4	25%	13.56	14
Medium Knowledge (11-17 points)	10	62.5%		
Good knowledge (≥17 points)	2	12.5%		

Statistical analysis was also conducted to verify the possible existence of associations/correlations between some of the variables under study or differences between IT and NT. This analysis was conducted individually for each of the groups (IT and NT). Metabolic control was categorized as a dichotomous categorical variable (adequate control if HbA1C ≤7%; inadequate control if HbA1C >7%), as well as disease duration (diagnosis less than 10 years ago or diagnosis 10 or more years ago). Responses to the DKT questionnaire were categorized as nominal categorical variables according to the “good, average, scarce” stratification, as previously described. The application of the Mann-Whitney test demonstrated statistically significant differences regarding the metabolic control (as a continuous numerical variable) of insulin-treated users when compared to non-insulin-treated users (p-value <0.05), favoring better metabolic control in the non-insulin-treated group. Regarding the degree of knowledge of the disease (according to DKT results), there was no statistically significant difference, using the Fisher's test (p-value = 0.20), between the two groups of diabetics studied. In the group of non-insulin-treated diabetics, there was no statistically significant association when applying the Fisher's test (p-value = 0.69) when evaluating metabolic control (as a dichotomous categorical variable) and the degree of knowledge according to the DKT result. The hypothesis of an association between metabolic control and the duration of the disease was tested in this group, and a statistically significant association was found using the Fisher's test (p-value = 0.029; odds ratio 3.83) between a longer duration of the disease and worse metabolic control.

In the population of insulin-treated diabetics, the authors evaluated the possible correlation between metabolic control and the result obtained in the DKT questionnaire, categorizing metabolic control as a dichotomous variable and the degree of knowledge by obtaining an evaluation of the questionnaire (good, average, scarce), by applying the Fisher's test, but it did not prove a statistically significant association (p-value = 0.62). The Fisher's test also demonstrated a statistically significant association (p-value = 0.025), in this group between the time of disease evolution and metabolic control, demonstrating an association between the longer time of disease evolution and inadequate metabolic control.

## Discussion

The results of this study provide important insights into the sociodemographic and clinical profile of patients with diabetes, as well as their level of knowledge about the disease, measured using the DKT. In general, the typical patient was a male, aged around 65-66 years, married or in a civil partnership, and with low educational attainment (up to primary school). Most participants (84%) were married, and more than half (59%) were retired. These findings reflect the typical characteristics of an aging population, where the diagnosis of diabetes, particularly DM2, becomes more prevalent. Indeed, 97% of participants had DM2, with most having lived with the condition for more than 10 years.

The analysis of the insulin-treated and non-insulin-treated groups revealed interesting differences. For instance, insulin-treated patients, compared to non-insulin-treated ones, showed a higher median BMI (28.1 vs. 27) and higher levels of HbA1C median (8.2% vs. 6.3%), indicating poorer metabolic control in the former group. This difference in metabolic control was supported by statistical analysis, which found a significant difference between the two groups, favoring the non-insulin-treated group in terms of glycemic control (p <0.05).

Regarding the level of knowledge about diabetes, measured by the DKT scale, both groups displayed a medium level of knowledge (59% for the non-insulin-treated and 62.5% for the insulin-treated). The application of this questionnaire showed that these diabetics had a sub-optimal knowledge regarding nutritional information in terms of which foods or drinks have higher carbohydrate content and their effect on blood glucose levels; the correct time interval that HbA1C represents average blood glucose levels; the effect of sickness or infection in blood sugar; and generally some difficulties identifying known complications associated with diabetes, such as the need to check and take care regularly of the feet, nerve disease, and signs of ketoacidosis.

The results we obtained by implementing the DKT are aligned with those found in the literature [11,13]. However, no statistically significant difference was found between the groups in terms of knowledge level (p = 0.20) in our study. This result suggests that insulin use alone does not influence patients' knowledge of the disease, although the lack of more advanced knowledge may be concerning as only a small proportion of both groups exhibited "good knowledge" (4% of non-insulin-treated and 12.5% of insulin-treated patients).

Additionally, the study found a significant association between the duration of the disease and metabolic control, both in the non-insulin-treated group (p = 0.029) and in the insulin-treated group (p = 0.025). In both cases, it was observed that a longer duration of diagnosis was associated with poorer metabolic control, which may be related to the natural progression of diabetes and the challenges in maintaining adequate glycemic control over time in DM2. This finding highlights the need for continuous and personalized strategies for managing the disease as it progresses [14,15].

Other researchers reported correlations between better knowledge of disease and sociodemographics variables (such as age, longer duration of diabetes, or higher education levels), which were not found in the present study [12,16-20]. In the study of Azevedo and Santiago (2016), a correlation between a better DKT score and better metabolic control was also not noted in our results [13]. Literature supports that lower levels of health literacy are associated with worse health outcomes, and the same applies to diabetes [2,9,21-25]. Our study showed a concerning level of knowledge about diabetes, measured by the DKT. Although this is not a measure of health literacy per se, we can imply that health literacy levels could also be low in our population, as it is the case in the general Portuguese population [26].

The authors encountered some limitations during this study. Firstly, the fact that it was only possible to implement this study recurring to a small convenience sample (n=99) could lead to difficulties in generalizing the results found. The authors would like to note that, in the future, it would be beneficial to reproduce this study with a proper representative and randomized sample, with a higher number of individuals. The health units included in this study are part of a more urban area in Portugal. The study was performed in Braga, which is one of the biggest cities in Portugal, with a strong economy and industry. The inclusion of other areas, which could be representative of urban and rural areas, across the country would be ideal. The organizational model of the health units included are among the most organized types that can be found in Portugal, so it would also be interesting to include units with other organizational models. The authors also had some resource limitations regarding human, time, and financial resources that limited the scope of this study. The authors would also like to point out that this being a cross-sectional study only helps in getting a perspective of diabetes knowledge among the diabetics included. It would be relevant to design a study with an intervention focusing on improving basic knowledge about nutrition, the disease (its signs, symptoms, complications, follow-up, treatment, and general management), and health literacy and try to measure changes in diabetes knowledge and metabolic control over time. The authors idealize that said interventions could occur on different levels: on a day-to-day basis during nursing and medical appointments, emphasizing this information about the disease; implementing an institutional campaign to improve diabetes knowledge and health literacy; promoting discussions within the institution about the results and the need for improvement; raising awareness among health staff and providing them with training to intervene and assist the diabetics; organizing small lectures by health professionals for diabetics to discuss and provide information on these topics; and disseminating this information through various channels within the institution to reach patients (flyers, posters, newsletters, informative bulletins, social media, and others). Lastly, the DKT questionnaire has been updated and a revised version (DKT2) is now available; however, it has not yet been translated and validated for the Portuguese population [10]. 

## Conclusions

This study provides a comprehensive analysis of the characteristics of patients with diabetes and the factors affecting both metabolic control and disease knowledge. While most participants showed adequate glycemic control, particularly those not treated with insulin, a sizable portion still exhibited suboptimal control, especially among patients with a longer disease duration. Although general knowledge of diabetes was classified as "medium" for most patients, this level of knowledge may not be sufficient to ensure good self-care, especially in more vulnerable groups, such as insulin-treated patients and those with a longer duration of disease. The lack of a significant difference in knowledge between these groups indicates that targeted educational interventions are necessary to enhance diabetes management and prevent long-term complications.

The association between the duration of diagnosis and poorer metabolic control in both groups underlines the importance of rigorous and ongoing follow-up of patients, with a focus on personalized treatment strategies and health education. This finding underscores the importance of early and sustained interventions to mitigate the progression of diabetes and its associated complications. Furthermore, these results emphasize the need for public health policies that promote enhanced diabetes education, aiming to empower patients to manage their condition more effectively, particularly in the advanced stages of the disease and among those with poorer metabolic outcomes.
